# Carfilzomib alters the HLA-presented peptidome of myeloma cells and impairs presentation of peptides with aromatic C-termini

**DOI:** 10.1038/bcj.2016.14

**Published:** 2016-04-08

**Authors:** D J Kowalewski, S Walz, L Backert, H Schuster, O Kohlbacher, K Weisel, S M Rittig, L Kanz, H R Salih, H-G Rammensee, S Stevanović, J S Stickel

**Affiliations:** 1 Department of Immunology, Institute for Cell Biology, University of Tübingen, Tübingen, Germany; 2 Department of Hematology and Oncology, University of Tübingen, Tübingen, Germany; 3 Applied Bioinformatics, Department of Computer Science, Center for Bioinformatics, University of Tübingen, Tübingen, Germany; 4 Quantitative Biology Center, University of Tübingen, Tübingen, Germany; 5 Max Planck Institute for Developmental Biology, Tübingen, Germany; 6 Clinical Cooperation Unit Translational Immunology, German Cancer Consortium (DKTK), DKFZ Partner Site, Tübingen, Germany; 7 German Cancer Consortium (DKTK), DKFZ Partner Site, Tübingen, Germany

## Abstract

Recent studies suggest that multiple myeloma is an immunogenic disease, which might be effectively targeted by antigen-specific T-cell immunotherapy. As standard of care in myeloma includes proteasome inhibitor therapy, it is of great importance to characterize the effects of this treatment on HLA-restricted antigen presentation and implement only robustly presented targets for immunotherapeutic intervention. Here, we present a study that longitudinally and semi-quantitatively maps the effects of the proteasome inhibitor carfilzomib on HLA-restricted antigen presentation. The relative presentation levels of 4780 different HLA ligands were quantified in an *in vitro* model employing carfilzomib treatment of MM.1S and U266 myeloma cells, which revealed significant modulation of a substantial fraction of the HLA-presented peptidome. Strikingly, we detected selective down-modulation of HLA ligands with aromatic C-terminal anchor amino acids. This particularly manifested as a marked reduction in the presentation of HLA ligands through the HLA allotypes A*23:01 and A*24:02 on MM.1S cells. These findings implicate that carfilzomib mediates a direct, peptide motif-specific inhibitory effect on HLA ligand processing and presentation. As a substantial proportion of HLA allotypes present peptides with aromatic C-termini, our results may have broad implications for the implementation of antigen-specific treatment approaches in patients undergoing carfilzomib treatment.

## Introduction

Proteasome inhibitors have become a cornerstone in the management of multiple myeloma (MM), effectively helping to increase disease-free and overall survival of MM patients over the past decade.^1^ Carfilzomib, a second-generation proteasome inhibitor, has been approved for patients with relapsed or refractory disease who have received at least two prior therapies and is currently under investigation as a first-line therapeutic option.^2, 3, 4^ By specifically and irreversibly binding to the β_5_-subunit, carfilzomib blocks the chymotrypsin-like specificity of the proteasome resulting in the activation of pro-apoptotic and anti-proliferative pathways^5, 6^ and the induction of a terminal unfolded protein response.^7^ As the proteasome has a central role in the generation of MHC-presented peptides,^8, 9, 10^ it has long been established that proteasome inhibition can directly impact antigen presentation by MHC molecules and thereby impair specific T-cell responses.^11, 12, 13^ In MM, the presence of clonally expanded CD8^+^ T cells has been associated with improved patient survival, pointing to their involvement in tumor surveillance.^14, 15^ Furthermore, the clinical efficacy of the immune modulatory drug lenalidomide,^16^ which has pleiotropic effects including improved cytotoxic T-cell activation,^17^ indicates the potentially central role of myeloma-specific T cells in disease control. In a recent study, we investigated the underlying specificities of anti-myeloma T-cell responses by analyzing the antigenic landscape of MM by mass spectrometry and identified a set of antigens characterized by exquisite myeloma specificity.^18^ As MM remains a largely incurable disease despite the aforementioned advances,^19, 20^ the aim of our previous study was to define a panel of broadly presented targets for antigen-specific immunotherapy of MM. Since standard of care in MM comprises proteasome inhibitor therapy, it is of great importance to thoroughly characterize the effects of this treatment on the antigenic landscape of myeloma cells to allow for implementation of robustly presented targets for concomitant or subsequent immunotherapy. In the present study, we comprehensively and semi-quantitatively mapped the impact of proteasome inhibition on HLA-restricted antigen presentation using an *in vitro* model of carfilzomib treatment in myeloma. Quantitation of the presentation levels of 72 previously defined myeloma antigens under treatment identified robustly presented targets. Importantly, peptidome-wide analysis delineated clusters of HLA ligands characterized by substantial and sustained down-modulation upon proteasome inhibition. Closer investigation of these clusters revealed distinct peptide motif-specific inhibitory effects of carfilzomib on HLA-restricted antigen presentation, which manifested as the marked reduction in the presentation of antigens with aromatic C-termini.

## Materials and methods

### Patients and bone marrow samples

Bone marrow mononuclear cells from MM patients at the time of diagnosis or at relapse before therapy were isolated by density gradient centrifugation (Biocoll, Biochrom GmbH, Berlin, Germany) and erythrocyte lysis (EL buffer, Qiagen, Venlo, Netherlands). Informed consent was obtained in accordance with the Declaration of Helsinki protocol. The study was performed according to the guidelines of the local ethics committee (142/2013BO2). Patient characteristics are provided in Supplementary Table 1.

### Myeloma cell lines

For HLA quantification and HLA ligandome analysis, the myeloma cell lines (MCLs) MM.1S, U266, RPMI8226 and JJN3 were cultured in the recommended cell media (RPMI-1640; Gibco, Carlsbad, CA, USA and IMDM; Lonza, Basel, Switzerland) supplemented with fetal calf serum, 100 IU/l penicillin, 100 mg/l streptomycin and 2 mmol/l glutamine at 37 °C and 5% CO_2_.

### *In vitro* treatment of MCL and primary MM samples

Cultured MCLs (MM.1S and U266) and primary myeloma samples were incubated with carfilzomib (100 nm) for a 1-h period, followed by three washes in PBS (Gibco) and recultured for additional 24 or 48 h. Controls were incubated with vehicle control (glucose 5%) for 1 h, followed by identical washing and incubation for 24 or 48 h. Experiments were conducted in three biological replicates where indicated. Please note that one data set (Carfilzomib #2 *t*_24 h_) did not pass quality control for label-free quantitation (LFQ) analysis and had to be replaced by Carfilzomib #1 *t*_24 h_. All analyses based on LFQ data therefore implement Carfilzomib #1 compared with Mock #2 as the data set for carfilzomib-induced modulation at *t*_24h_.

### Quantification of HLA surface expression

HLA surface expression on CD38^+^CD138^+^ myeloma cells of patients and MCLs was analyzed using the QIFIKIT bead-based quantitative flow-cytometric assay (Dako, Glostrup, Denmark) according to the manufacturer's instructions as described before.^21^ In brief, samples were stained with the pan-HLA class I-specific monoclonal antibody (mAb) W6/32 (produced in-house) or IgG isotype control (BioLegend, San Diego, CA, USA), respectively. Surface marker staining for primary samples was carried out with directly labeled CD138, anti-κ, anti-λ, CD19, CD20 (BioLegend) and CD38, CD3 and CD34 (BD, Franklin Lakes, NJ, USA) antibodies. 7-AAD (BioLegend) was added as a viability marker immediately before flow-cytometric analysis on an LSR Fortessa (BD).

### Isolation of HLA ligands from MCLs

HLA class I molecules were isolated using standard immunoaffinity purification as described before,^22^ using the pan-HLA class I-specific mAb W6/32 (produced in-house) to extract HLA ligands from the fixed sample volume of 2.0 ml cell pellet per condition and biological replicate.

### Analysis of HLA ligands by LC-MS/MS

HLA ligand extracts were analyzed in five technical replicates as described previously.^23^ In brief, peptide samples were separated by nanoflow HPLC (RSLCnano, Thermo Fisher, Waltham, MA, USA) using a 50 μm × 25 cm PepMap RSLC column (Thermo Fisher) and a gradient ranging from 2.4 to 32.0% acetonitrile over the course of 90 min. Eluting peptides were analyzed in an online coupled LTQ Orbitrap XL mass spectrometer (Thermo Fisher) using a top five CID (collision-induced dissociation) fragmentation method.

### Database search and HLA annotation

Data processing was performed as described previously.^23^ In brief, the Mascot search engine (Mascot 2.2.04; Matrix Science, London, UK) was used to search the human proteome as comprised in the Swiss-Prot database (20 279 reviewed protein sequences, 27 September 2013) without enzymatic restriction. Oxidized methionine was allowed as a dynamic modification. The false discovery rate was estimated using the Percolator algorithm^24^ and set to 5%. Peptide lengths were limited to 8–12 amino acids for HLA class I. Protein inference was disabled, allowing for multiple protein annotations of peptides. HLA annotation was performed using NetMHC^25^ (v3.4), annotating peptides with IC_50_ scores below 500 nm as ligands of the corresponding HLA allotype. In cases of multiple possible annotations, the HLA allotype yielding the lowest IC_50_ score was selected.

### LFQ of HLA ligand presentation

For LFQ of the relative HLA ligand abundances over the course of carfilzomib treatment, the total injected peptide amounts of all samples were normalized and LC-MS/MS analysis was performed in five technical replicates for each sample. For normalization, the relative amounts of substance in the different samples/conditions were calculated from the summed areas under the curve of all peptide identifications detected in dose-finding mass spectrometry runs and the samples were adjusted accordingly by dilution. Relative quantification of HLA ligands was performed by calculating the area under the curve of the corresponding precursor extracted ion chromatograms (XIC) using ProteomeDiscoverer 1.4 (Thermo Fisher). For Volcano plots, the ratios of the mean areas of the individual peptides in the five LFQ-MS runs of each sample were calculated and two-tailed *t*-tests implementing Benjamini–Hochberg correction were performed using an in-house R script (v3.2).

### Software and statistical analysis

Flow-cytometric data analysis was performed using FlowJo 7.2 (Treestar, Ahland, OR, USA). An in-house Python script was used for permutation analysis in the calculation of false discovery rates of treatment-associated peptide presentation, as described previously.^23^ In-house R scripts were utilized for volcano plots and longitudinal analysis of relative HLA ligand abundances.

## Results

### The impact of carfilzomib treatment on HLA surface expression of myeloma cells is heterogeneous and transient

**Figure 1:**
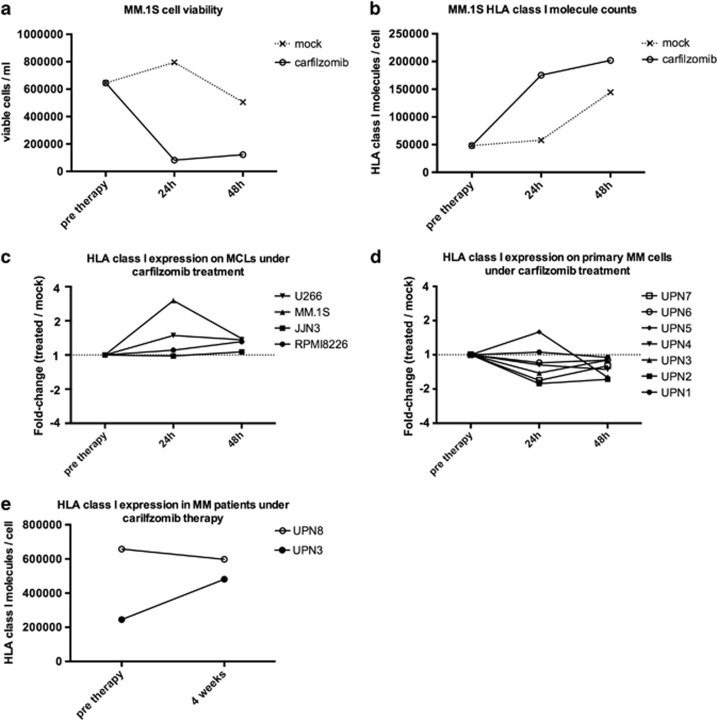
Effects of carfilzomib on myeloma cell viability and HLA class I surface expression. Quantification of HLA surface expression was performed using a bead-based flow-cytometric assay using the pan-HLA class I-specific monoclonal antibody W6/32. (**a**) Viability of MM.1S cells before *in vitro* treatment and 24 h/48 h after a 1-h pulse with 100 nm carfilzomib (Carfilzomib) or 5% glucose (MOCK). Cell viability was determined using the trypan blue exclusion test. (**b**) Absolute counts of HLA class I surface molecules on MM.1S cells under *in vitro* treatment. (**c**) Longitudinal analysis of relative, mock-normalized changes in HLA class I surface expression on four different MCLs under *in vitro* treatment. (**d**) Longitudinal analysis of relative, mock-normalized changes in HLA class I surface expression on primary CD38^+^CD138^+^ myeloma cells from seven different patients (UPN1-7) under *in vitro* treatment. (**e**) Absolute counts of HLA class I surface molecules on primary CD38^+^CD138^+^ myeloma cells from bone marrow aspirate of two different patients before commencement of therapy and after 4 weeks of treatment with carfilzomib.

To assess the impact of proteasome inhibition on HLA surface expression, we performed longitudinal quantification of surface HLA class I molecule counts on MCLs and primary myeloma cells treated with carfilzomib. First, we analyzed the cytotoxicity of treatment with carfilzomib on MCLs and detected a steep decline in viability of MM.1S cells as early as 24 h after treatment (*t*_24h_, −87.3%) followed by a stabilization of the surviving population at *t*_48h_ (Figure 1a). To account for effects of sample handling on HLA surface expression, we directly compared carfilzomib-treated cells with mock-treated controls (Figure 1b). Comparing the normalized HLA class I expression kinetics of four MCLs (U266, MM.1S, JJN3 and RPMI8226), we observed heterogeneous effects on surface HLA exposure with changes in molecule counts ranging from virtually no regulation (−1.9%, JJN3) to threefold increases (MM.1S, +201.8%) at *t*_24h_. At *t*_48h_, the effects of treatment ranged from +6.6% (JJN3) to +39.8% (MM.1S) increase in surface HLA class I (Figure 1c). Investigating the impact of *in vitro* treatment on primary myeloma cells we generally observed down-modulation of surface HLA class I with 5/7 samples showing decreases ranging from −14.9% to −44.3% at *t*_24h_. For 2/7 samples, we detected increased surface HLA expression of 5.5 and 59.1%, respectively. These effects were found to decline at *t*_48h_ with HLA levels compared baseline before treatment decreased by −5.0 to -39.1% (Figure 1d). To investigate the physiological effects of proteasome inhibitor therapy more closely, we analyzed HLA expression *ex vivo* on bone marrow-derived myeloma cells of two MM patients before therapy and after 4 weeks of carfilzomib treatment. In the patient showing higher levels of HLA class I at baseline (UPN8, 660 000 molecules/cell), we detected a slight down-modulation (−9.1%) after therapy. A second patient, who presented with 245 000 HLA class I molecules per cell at baseline, showed considerable up-modulation (UPN3, +96.2%) after treatment (Figure 1e). Taken together, we observed a high degree of plasticity and patient/cell line individuality in HLA surface expression upon carfilzomib treatment. Importantly, we did not observe loss of HLA class I expression, with the lowest detected surface molecule counts after treatment ranging at 190 000 molecules/cell (UPN2, *t*_24h_).

### Mass spectrometry enables semi-quantitative analysis of HLA class I restricted peptide presentation on MCLs under proteasome inhibitor therapy

**Figure 2:**
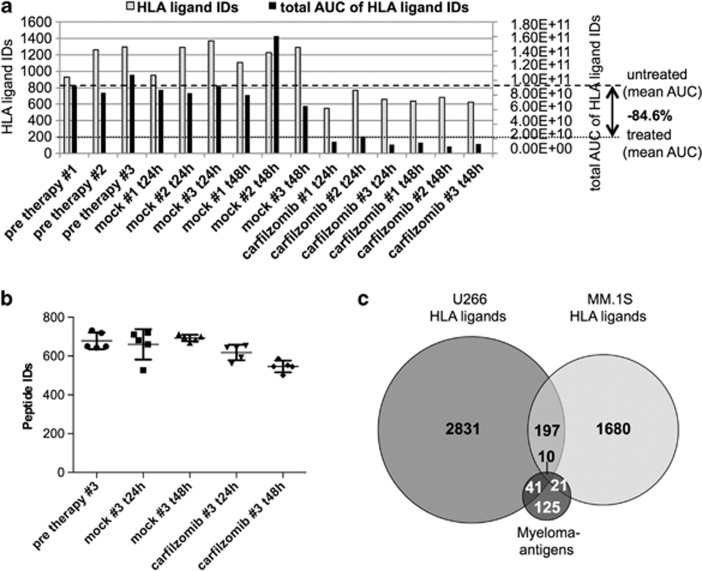
Mass spectrometric analysis of the HLA-presented peptidome of MM.1S cell under carfilzomib treatment. (**a**) HLA class I ligand extracts of MM.1S cells before *in vitro* treatment and 24 h/48 h (*t*_24h_, *t*_48h_) after a 1-h pulse with 100 nm carfilzomib (Carfilzomib) or 5% glucose (Mock) were analyzed in biological triplicates using sample shares of 20% in dose-finding mass spectrometry runs. The raw number of HLA ligand identifications and the summed area under the curves (AUC) of their extracted ion chromatograms are indicated in gray and black bars, respectively. (**b**) Numbers of peptide identifications after adjustment of the injected sample amounts. The sample amounts were adjusted according to their ratios of summed AUC and each condition was analyzed in five technical replicates, allowing for LFQ of the relative peptide abundances. (**c**) Overlap of the 1908 different HLA class I ligands identified on MM.1S cells compared with a set of 197 different myeloma-associated peptides described in an earlier study.^18^

To assess changes in HLA ligandome composition, our study employed a discovery and validation set design, utilizing MM.1S to define effects of carfilzomib and U266 to validate these findings in an independent set of samples. Direct mass spectrometric analysis of HLA ligand extracts was performed for biological triplicates of MM.1S cells before treatment and at *t*_24h_ and *t*_48h_, as well as for the corresponding mock-treated controls. Using the summed peptide intensities of all HLA ligand identifications in each condition as an indirect measure of total peptide abundance, we detected an 84.6% decrease in total HLA ligand presentation on treated cells (*t*_24h_ and *t*_48h_ combined) compared with levels before treatment or mock-treated controls (Figure 2a). To adjust the amount of peptide injected per LC-MS run across all samples, HLA ligand extracts of each condition were diluted according to their ratios of summed peptide intensities to the lowest yielding sample and LFQ was conducted in five technical replicates, yielding comparable numbers of peptide identifications (Figure 2b). In total, 2575 different HLA class I peptides were identified. Of these, 1908 (74.1%) peptides were computationally assigned to be binders of one of the MM.1S HLA allotypes (A*23:01, A*24:02, B*18:01, B*42:01, C*12:03, (C*17:01)), as defined by NetMHC scores of IC_50_<500 nm. As no NetMHC predictors were available for HLA-C*17:01, peptides restricted by this allotype had to be excluded from further analyses. Longitudinal quantification of HLA ligand presentation across all conditions and time points could be performed for a subset of 1068 HLA ligands. Importantly, 31 of the HLA ligands monitored on MM.1S cells were found to be highly specific myeloma-associated peptides identified in an earlier study by our group (Figure 2c, Supplementary Table 2).^18^ For U266, a similar LFQ strategy was implemented, analyzing HLA peptidome composition in triplicates of treated cells at *t*_24h_ and *t*_48h_ compared with a single baseline control before treatment. In total, 3730 different HLA class I peptides were identified, 3079 (82.6%) of which were computationally assigned to be binders of one of the U266 HLA allotypes (A*02:01, A*03:01, B*07:02, B*40:01, C*07:02, (C*03:04 not available)). A set of 51 previously established myeloma-associated peptides was identified on U266 (Figure 2c, Supplementary Table 3). Together, our discovery and validation data sets were designed to enable highly confident detection of modulations in HLA ligandome composition in two independent samples and furthermore allowed us to directly and quantitatively track the presentation of 72 potential therapeutic targets in this *in vitro* model of proteasome inhibitor therapy.

### Carfilzomib induces substantial qualitative and quantitative alterations in HLA class I peptide presentation

**Figure 3:**
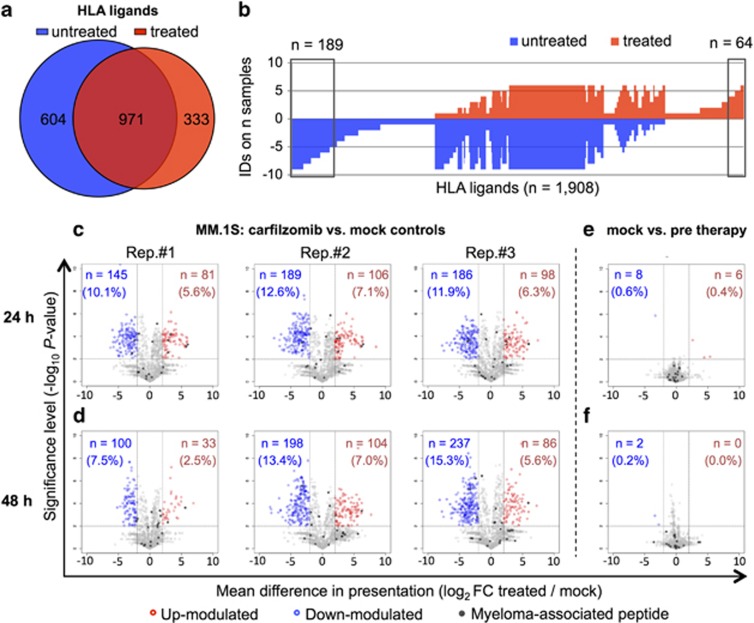
Carfilzomib induces substantial qualitative and quantitative changes in the HLA ligandome of MM.1S cells. (**a**) Overlap analysis of HLA class I ligands identified on carfilzomib-treated versus untreated (mock controls and pre-therapy) cells. (**b**) Frequency-based analysis of peptide presentation on treated versus untreated MM.1S cells. HLA ligands are indicated on the x axis and the numbers of samples on which they were detected on the y axis. The box on the right highlights HLA ligands showing significant treatment-associated presentation (exclusive presentation on ⩾4/6 treated samples, FDR=3.0%), whereas the box on the left indicates peptides lost after carfilzomib therapy (exclusive presentation on ⩾5/9 untreated samples, FDR=4.9%). (**c**–**f**) Volcano plots of modulations in the relative abundances of HLA ligands on MM.1S cells comparing the conditions indicated. Each dot represents a specific HLA ligand. Log2-fold changes of their abundance are indicated on the x axis and the corresponding significance levels after Benjamini–Hochberg correction on the y axis. HLA ligands showing significant up- or down-modulation (>4-fold change in abundance with *P*<0.01) are highlighted in red and blue, respectively. The numbers and percentages of these significantly modulated ligands are specified in the corresponding quadrants. (**c**, **d**) Volcano plots comparing HLA ligand abundances on carfilzomib versus mock-treated cells at *t*_24h_ and *t*_48h_, respectively. (**e**, **f**) Control volcano plots comparing HLA ligand abundances on mock-treated cells at *t*_24h_ and *t*_48h_ to baseline levels before therapy.

Overlap analysis revealed considerable qualitative differences in the HLA ligandome composition of untreated versus carfilzomib-treated MM.1S cells. Comparison of the nine untreated samples (three biological replicates each of MM.1S cells before treatment, and at *t*_24_ and *t*_48_ of mock treatment) with the six treated samples revealed 31.7% (604 HLA ligands) of the total MM.1S HLA ligandome to be exclusively presented on untreated cells, whereas 17.5% (333 HLA ligands) were only detectable after treatment with carfilzomib (Figure 3a). To control for the technical variability of LC-MS based peptide analysis, we plotted the rates of peptide detection in the two different conditions and calculated the significance thresholds for treatment-associated presentation of HLA ligands based on random permutation analysis as described previously (Supplementary Figure 1).^23^ Out of the 333 HLA ligands exclusively presented on treated MM.1S cells, 64 different HLA class I ligands (3.4% of the total MM.1S HLA ligandome, 67 source proteins) were found to be significantly associated with carfilzomib treatment (*P*<0.05, Figure 3b). Functional annotation clustering of the corresponding source proteins using the online bioinformatics resource DAVID^26^ for KEGG pathway analysis identified a single, small cluster (6/67 source proteins) of ribosomal proteins to be significantly enriched (11.7-fold enrichment, *P*<0.01, data not shown). No unifying characteristics were identified for the other 61 proteins using KEGG pathway or gene ontology analysis for biological processes (GO term BP FAT analysis).

Implementing LFQ data, we then quantitatively assessed HLA class I ligand presentation during proteasome inhibitor therapy. We observed considerable plasticity of the HLA class I ligandome after treatment with carfilzomib with 17.9±1.1% of MM.1S ligands and 11.2±0.7% of U266 ligands (mean of three biological replicates±s.d.) showing significant modulation (fold-change ⩾4, *P*<0.01 after Benjamini–Hochberg correction) at *t*_24h_ compared with mock-treated controls (Figure 3c, Supplementary Figure 2a). At *t*_48h_ similar proportions of modulation were observed with 17.1±5.0% (MM.1S) and 14.0±2.6% (U266) of HLA ligands significantly altered in their abundance (Figure 3d, Supplementary Figure 2b). Plotting HLA ligand presentation of mock-treated MM.1S controls at *t*_24h_ and *t*_48h_ compared with the levels of untreated cells before therapy yielded virtually no modulated ligands, which strongly indicates that the observed plasticity is a carfilzomib-induced effect (Figures 3e and f). Notably, for MM.1S 14/31 (45%) of the previously established myeloma-associated peptides were subject to carfilzomib-induced modulation in at least one biological replicate, with the majority showing up-modulation (10/14) compared with only 4/14 down-modulated ligands (Supplementary Table 2). For U266, we detected modulation for 13/51 (26%) of myeloma-associated peptides, with comparable fractions showing up- or down-modulation (up: 7/51, down 6/51, Supplementary Table 3).

**Figure 4:**
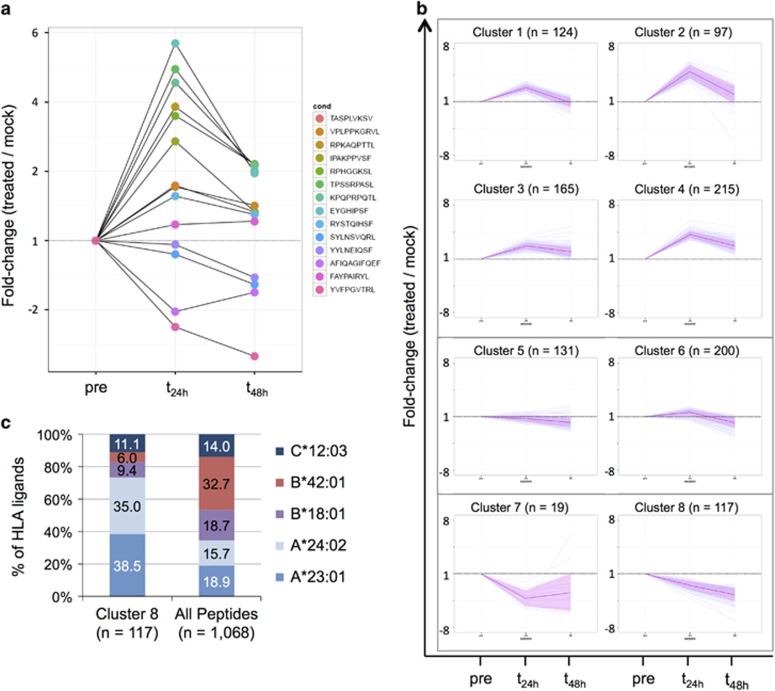
Kinetics of HLA class I ligand presentation on MM.1S cells after treatment with carfilzomib. (**a**) Longitudinal analysis of the presentation of 14 highly specific myeloma-associated peptides on MM.1S cells after treatment with carfilzomib. Modulations in peptide abundance are indicated on the y axis as fold-change compared with mock-treated controls. (**b**) Clustering of 1068 different HLA class I ligands according to their presentation kinetics upon treatment with carfilzomib. Modulations in relative peptide abundance are indicated on the y axis as fold-change compared with mock-treated controls. (**c**) Distribution of HLA restrictions of peptides represented in the down-modulated cluster 8 compared with the distribution of all 1068 tracked peptides.

To investigate the kinetics and duration of these carfilzomib-induced effects in greater detail, we used the MM.1S model to longitudinally track the abundances of the 14/31 myeloma peptides for which quantitative information was available across all time points and conditions. For the majority of these targets (10/14, 71.4%), we observed a peak in modulation at *t*_24h_ followed by a gradual decline toward baseline levels at *t*_48h_ (Figure 4a). Only 4/14 peptides (28.6%) showed persistent modulation even at *t*_48h_, with three of them showing progressive down-modulation after treatment. To relate these findings to the general kinetics of HLA ligand presentation on MM.1S cells after carfilzomib treatment, we plotted the relative abundances of the 1068 peptides, which could be quantified longitudinally and performed unsupervised clustering to identify peptides with similar modulation patterns. Eight different clusters were identified based on intra-cluster sum of squares analysis. Four clusters (601 of 1068 tracked peptides, 56.3%) were found to comprise peptides characterized by transient up-modulation of different degrees at *t*_24h_ followed by partial or total reversion to baseline levels at *t*_48h_ (Figure 4b). Only 2/8 clusters displayed considerable down-modulation, with only cluster 8 showing persistent down-modulation even 48 h after carfilzomib treatment. Functional annotation clustering of the 126 source proteins represented in cluster 8 did not reveal any significant enrichment of specific KEGG pathways. However, strikingly, analysis of HLA restriction patterns revealed a distinct enrichment of peptides restricted by the HLA allotypes A*23:01 and A*24:02 in this down-modulated cluster (86/117 peptides, 73.5%) compared with their overall frequency (370/1068, 36.4%, Figure 4c). As both these HLA allotypes are characterized by a virtually identical binding motif,^27^ their selective down-modulation suggested either allotype- or peptide motif-specific effects of carfilzomib on HLA ligandome composition.

### Carfilzomib alters HLA class I ligand presentation in an HLA allotype-specific manner

**Figure 5:**
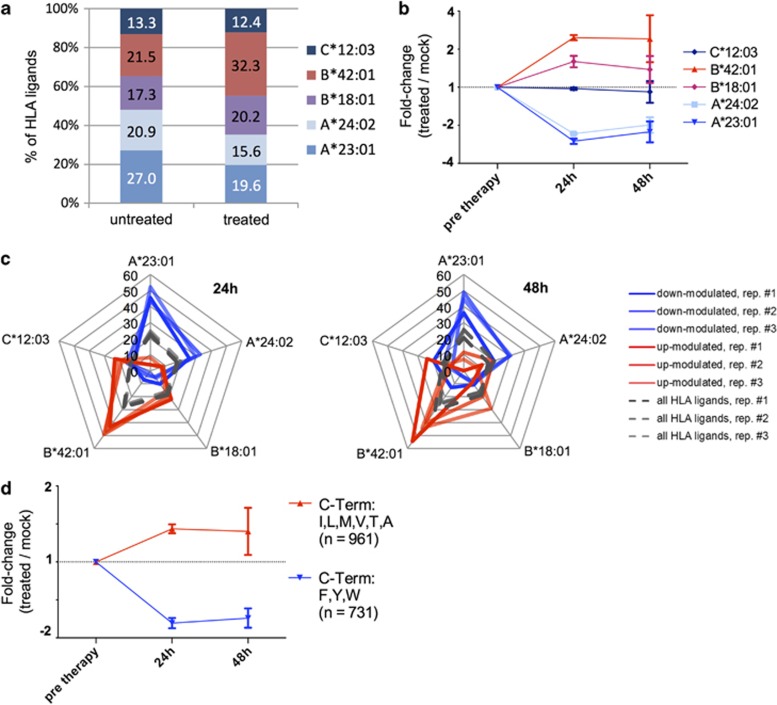
Carfilzomib alters HLA class I restricted peptide presentation in an HLA allotype-specific manner. (**a**) Distribution of HLA restrictions among peptides identified on carfilzomib-treated (*n*=1575 peptides) versus untreated MM.1S cells (*n*=1,304 peptides). (**b**) Longitudinal analysis of HLA ligand abundances after carfilzomib treatment grouped according to HLA restrictions. HLA allotype-specific fold-change values were calculated as the mean fold-change of all peptides restricted by the respective allotype. Data points represent mean fold-change values of three biological replicates±s.d. (**c**) Radar plots of the distribution of HLA restrictions among peptides showing significant down-modulation (blue lines) or up-modulation (red lines) compared with the distribution among all HLA ligands (gray dashed lines). Radar plots consist of overlays of three biological replicates. (**d**) Longitudinal analysis of HLA ligand abundances after carfilzomib treatment dichotomized according to their C-terminal anchor amino-acid groups. Data points represent mean fold-change values of three biological replicates±s.d.

To systematically investigate the impact of carfilzomib treatment on antigen presentation through specific HLA allotypes, we first analyzed the overall frequencies of HLA allotype restrictions of ligands on treated versus untreated myeloma cells. For MM.1S, this comparison based on qualitative data (Figure 3a) confirmed an overall decrease in the frequencies of HLA ligand identifications annotated with HLA-A*23:01 (−5.3%) and A*24:02 (−7.4%) on treated MM.1S cells (Figure 5a). This effect was accompanied by a substantial increase for HLA-B*42:01 (+10.8%), a small increase for B*18:01 (+2.9%) and virtually no change in the HLA ligand identification rate for C*12:03 (+0.9%). For U266 slight alterations in the frequencies of peptides restricted by HLA-A*02:01 (+2.1%) and A*03:01 (−2.0%) were detected (Supplementary Figure 3a). As mass spectrometry enables peptide identification across a large dynamic range of abundance, we abandoned HLA restriction analysis based on qualitative data in favor of a quantitative approach: by analyzing the restriction patterns of HLA ligands showing significant quantitative modulation upon treatment (Figures 3c and d, Supplementary Figures 2A and B), we detected substantial and very distinct distortions in the HLA allotype distribution of MM.1S: at *t*_24h_ 47.0±3.8% and 29.3±3.0% of down-modulated HLA ligands were restricted by HLA-A*23:01 and A*24:02, respectively, together accounting for the vast majority (76.3%) of down-modulated ligands (Figure 5c). Concomitantly, the majority of up-modulated HLA ligands was restricted by HLA-B*42:01 (46.4±2.4%). These distortive effects persisted at *t*_48h_, albeit with considerably larger deviations between the biological replicates. For U266, we observed only slightly distortive effects of treatment, resulting mainly in a moderate enrichment of peptides restricted by HLA-B*07:02 among down-modulated ligands at *t*_24h_ (37.3±2.0% compared with 32.4±0.1% among all ligands, Supplementary Figure 3C).

Next, we longitudinally plotted the abundances of peptides grouped by HLA restriction in order to determine and quantify the kinetics of allotype-specific effects of carfilzomib. On MM.1 S, we detected maximum down-modulation of peptides restricted by HLA-A*23:01 and A*24:02 at *t*_24h_ with peptide abundances reduced by −62.5±1.8% and −57.0±0.6%, respectively, followed by a very limited and slow recovery of peptide levels at *t*_48h_ (−54.7±9.0 and −49.2±7.0%, Figure 5b). This reduction was compensated by increased levels of HLA-B*42:01 (*t*_24h_: +147.1±11.5%, *t*_48h_: +163.4±103.6%) and B*18:01 (*t*_24h_: +60.5±17.1%, *t*_48h_: +42.2±31.6%). No substantial modulation was detected for HLA-C*12:03 ligands.

Of note, for U266 no allotype specificity of modulatory effects became evident (Supplementary Figure 3B), thus indicating that the underlying mechanisms of HLA ligand modulation may not be due to direct and differential regulation of HLA class I allotype expression. As proteasomal cleavage is thought to have the defining role in the generation of HLA ligand C-termini, we next investigated whether the observed allotype-specific modulation of HLA ligands could be explained by altered C-terminal amino-acid distribution. As carfilzomib selectively inhibits the chymotrypsin-like specificity of the β_5_-proteasomal subunit which mainly generates peptides with aromatic C-termini we dichotomized HLA ligands into two groups characterized by aromatic (F/Y/W) or aliphatic (I/L/M/V/T/A) C-terminal anchor amino acids. Strikingly, analysis of the presentation kinetics of these peptide groups indeed revealed a distinct reduction in the relative abundance of HLA ligands with aromatic C-termini after treatment, which was independently observed in both MCL model systems. In MM.1S, we detected a reduction of the aromatic group (*t*_24h_: −42.7±2.7%, *t*_48h_: −39.9±5.2%), which was offset by an increase in ‘aliphatic' HLA ligands (*t*_24h_: +35.5±5.6%, *t*_48h_: 35.4±29.4% Figure 5d). In U266, similar kinetics were observed with aromatic peptides reduced by −46.0±7.0% and −52.4±27.5% at *t*_24h_ and *t*_48h_, respectively. Together, these findings point toward an underlying peptide motif-specific effect of carfilzomib, which selectively impairs antigen presentation of peptide ligands with aromatic C-terminal amino acids.

## Discussion

Despite the central role of proteasome inhibitor therapy in the management of MM,^1^ systematic investigations regarding the impact of this treatment on antigen presentation have been scarce. The — to our knowledge — only previous mass spectrometry-based study systematically investigated the impact of first-generation proteasome inhibitors on protein turnover and antigen presentation in breast cancer cell lines and found evidence of complex mechanistic underpinnings, which prevented generalized conclusions and called for further investigation.^28^ With the breakthrough success of immune checkpoint blockade eliciting long-term disease control in solid tumor patients,^29, 30, 31, 32, 33^ the identification and characterization of antigens targeted by anti-cancer T-cell responses has re-entered the spotlight.^34, 35, 36^ Despite the — so far — underwhelming efficacy of checkpoint blocking antibodies in MM^37^ the effectiveness of another class of immune modulators^16, 38, 39^ and the association of clonally expanded CD8^+^ T cells with improved clinical outcome^14, 15^ indicates the relevance of immune surveillance in MM. Previous studies of our group which mapped the HLA-presented antigenic landscape of hematologic malignancies indicated that a mass spectrometry-based approach is highly efficient in identifying the physiological targets of such anti-cancer T-cell responses in leukemia patients.^23, 40^ In MM, this strategy enabled us to delineate a panel of highly specific myeloma-associated antigens, which may be implemented for antigen-specific immunotherapy.^18^ With carfilzomib being a frontrunner for the future first-line therapy of MM,^2, 3, 4^ the present study was designed to quantify the impact of this proteasome inhibitor on the HLA-presented antigenic landscape thereby allowing to pinpoint robustly presented targets for antigen-specific T-cell immunotherapy.

We utilized an established model of treatment applying a 1-h pulse of carfilzomib *in vitro*, which mimics the pharmacokinetics of this drug *in vivo*^41^ and was previously reported to result in 80% reduction of the chymotrypsin-like activity of the proteasome.^42^ This treatment resulted in heterogenic effects on the HLA surface expression of MCLs, which correlate with their reported sensitivity to proteasome inhibition.^7^ LFQ by mass spectrometry revealed that treatment-induced modulation of HLA ligand presentation on MCL cells upon proteasome inhibition was substantial, affecting an approximately sixfold greater proportion of the HLA ligandome than observed in similar experiments treating primary cells of chronic lymphocytic leukemia with the immunomodulatory drug lenalidomide (Nelde *et al.*, manuscript in preparation). In general, the effects of carfilzomib on HLA surface expression and HLA ligand presentation were transient with peak modulation typically detected 24 h after treatment followed by a total or gradual reversion to baseline levels after 48 h, which is in accordance with the recovery rate of proteasome activity described previously.^42^ Notably, the observed shift in HLA ligandome composition did not appear to be driven by the differential presentation of source proteins from specific pathways but rather seems to be the result of direct distortive effects of carfilzomib on HLA ligand generation. As these effects particularly manifested on MM.1 S cells as a marked reduction of HLA ligands restricted by HLA-A*23:01 and A*24:02, which are characterized by a virtually identical binding motif,^27^ we hypothesized that the observed down-modulation rather is a direct consequence of the inhibition of the chymotrypsin-like activity of the proteasome leading to the generation of peptides with altered C-terminal anchors.^43^ Dichotomization of MM.1S peptides according to their C-terminal residues indeed indicated the validity of this concept, but had to be interpreted with caution due to the interdependency of HLA restriction and the structure of the C-terminal anchor amino acid.^44^ However, the reproduction of these effects on U266 cells, which are characterized by a completely non-overlapping HLA type, further lends support to this hypothesis. Together, our findings suggest that carfilzomib can induce substantial qualitative and quantitative alterations in HLA ligandome composition on myeloma cells and selectively impairs the presentation of HLA ligands with aromatic C-termini. Further studies into the functional impact of these alterations on T cell-mediated immune recognition are needed to clarify the implications of these findings for cancer immunosurveillance in patients undergoing proteasome inhibitor therapy and may help to identify optimal regimens for antigen-specific immunotherapy for MM.
