# Supplementary material for: Carfilzomib alters the HLA-presented peptidome of myeloma cells and impairs presentation of peptides with aromatic C-termini
Source: Blood Cancer J. 2016 Apr 8;6(4):e411–. doi: 10.1038/bcj.2016.14 (PMC4855252; doi:10.1038/bcj.2016.14)
Supplement: Supplementary Figure Legends [file bcj201614x1.docx]

**Supplemental Figure Legends**

**Supplemental Figure 1.** **Definition of the thresholds for treatment-associated presentation of HLA ligands**

Based on random permutation analysis, the false discovery rates of carfilzomib-associated peptide presentation/loss were calculated for different cutoff values of detection, as described previously (23).

**Supplemental Figure 2.** **Carfilzomib induces substantial qualitative and quantitative changes in the HLA ligandome of U266 cells**

Volcano plots of modulations in the relative abundances of HLA ligands on U266 cells comparing the conditions indicated. Each dot represents a specific HLA ligand. Log2-fold-changes of their abundance are indicated on the x-axis, the corresponding significance levels after Benjamini-Hochberg correction on the y-axis. HLA ligands showing significant up- or down-modulation (> 4‑fold change in abundance with *P*< 0.01) are highlighted in red and blue, respectively. The numbers and percentages of these significantly modulated ligands are specified in the corresponding quadrants. (A, B) Volcano plots comparing HLA ligand abundances on carfilzomib treated versus pre-treateatment cells at t_24h_ and t_48h_, respectively. (C) Control volcano plots comparing HLA ligand abundances between different replicates at t_24h_ and t_48h_.

**Supplemental Figure 3.** **Effects of carfilzomib on HLA restriction patterns and ligand abundances on U266 cells**

(A) Distribution of HLA restrictions among peptides identified on carfilzomib-treated (n = 2,648 peptides) versus untreated U266 cells (n = 2,429 peptides). (B) Longitudinal analysis of HLA ligand abundances after carfilzomib-treatment grouped according to HLA restrictions. HLA allotype-specific fold-change values were calculated as the mean fold-change of all peptides restricted by the respective allotype. Data points represent mean fold-change values of 3 biological replicates ± SD. (C) Radar plots of the distribution of HLA restrictions among peptides showing significant down-modulation (blue lines) or up-modulation (red lines) compared to the distribution among all HLA ligands (gray dashed lines). Radar plots consist of overlays of 3 biological replicates. (D) Longitudinal analysis of HLA ligand abundances after carfilzomib-treatment dichotomized according to their C-terminal anchor amino acid groups. Data points represent mean fold-change values of 3 biological replicates ± SD.
