# Supplementary material for: Carfilzomib alters the HLA-presented peptidome of myeloma cells and impairs presentation of peptides with aromatic C-termini
Source: Blood Cancer J. 2016 Apr 8;6(4):e411–. doi: 10.1038/bcj.2016.14 (PMC4855252; doi:10.1038/bcj.2016.14)
Supplement: Supplementary Table 1 [file bcj201614x2.docx]

**Supplemental Table 1:**

Patient characteristics

| **UPN** | **Sex** | **Age** | **ISS Stage** | **Durie & Salmon** | **Light chain restriction** | **Cytogenetic risk** | **Previous therapy** |
| --- | --- | --- | --- | --- | --- | --- | --- |
| 1 | f | 72 | 2 | 3A | kappa | high | yes |
| 2 | m | 74 | 3 | 2B | kappa | standard | yes |
| 3 | m | 68 | 1 | 3A | kappa | unknown | yes |
| 4 | f | 70 | 1 | 1A | kappa | standard | no |
| 5 | f | 71 | 3 | 3A | kappa | high | yes |
| 6 | f | 71 | 1 | 3A | kappa | unknown | no |
| 7 | f | 53 | 3 | 3A | kappa | standard | no |
| 8 | f | 74 | 2 | 2A | kappa | standard | yes |

Abbreviations: UPN, uniform patient number; cytogenetic risk according to the IMWG risk stratification (Chng et. al. Leukemia 2014).
