# Supplementary material for: Carfilzomib alters the HLA-presented peptidome of myeloma cells and impairs presentation of peptides with aromatic C-termini
Source: Blood Cancer J. 2016 Apr 8;6(4):e411–. doi: 10.1038/bcj.2016.14 (PMC4855252; doi:10.1038/bcj.2016.14)
Supplement: Supplementary Table 2 [file bcj201614x3.docx]

| **Myeloma antigen** | **HLA** | **Source Protein** | **24#1** | **24#2** | **24#3** | **48#1** | **48#2** | **48#3** |
| --- | --- | --- | --- | --- | --- | --- | --- | --- |
| RYLDLFTSF | A*24:02 | KDELR2 | **-1** | **-1** | **-1** | 0 | **-1** | **-1** |
| AFIQAGIFQEF | A*23:01 | RAD1 | **-1** | 0 | **-1** | 0 | 0 | **-1** |
| SEFDFFERL | C*12:03 | SEMA4A | 0 | 0 | 0 | 0 | 0 | **-1** |
| YVFPGVTRL | C*12:03 | SPATC1L | 0 | 0 | 0 | **-1** | 0 | 0 |
| TFLPFIHTI | A*23:01 | BFAR | 0 | 0 | 0 | 0 | 0 | 0 |
| RYFKGPELL | A*24:02 | CSNK2A1 | 0 | 0 | 0 | 0 | 0 | 0 |
| RYSPVLSRF | A*24:02 | COG1 | 0 | 0 | 0 | 0 | 0 | 0 |
| RYSTQIHSF | A*24:02 | BHLHA15 | 0 | 0 | 0 | 0 | 0 | 0 |
| SYLNSVQRL | A*24:02 | NUPL2 | 0 | 0 | 0 | 0 | 0 | 0 |
| YYLNEIQSF | A*24:02 | SPATC1L | 0 | 0 | 0 | 0 | 0 | 0 |
| NEFPVFDEF | B*18:01 | MB21D1 | 0 | 0 | 0 | 0 | 0 | 0 |
| IPAKPPVSF | B*42:01 | TXNDC11 | 0 | 0 | 0 | 0 | 0 | 0 |
| RPHGGKSL | B*42:01 | TXNDC11 | 0 | 0 | 0 | 0 | 0 | 0 |
| RPQLKGVVL | B*42:01 | MRPS12 | 0 | 0 | 0 | 0 | 0 | 0 |
| SPALPGLKL | B*42:01 | TNFRSF13B | 0 | 0 | 0 | 0 | 0 | 0 |
| TPAVGRLEV | B*42:01 | CDCA8 | 0 | 0 | 0 | 0 | 0 | 0 |
| FAQIISVALI | C*12:03 | DOLK | 0 | 0 | 0 | 0 | 0 | 0 |
| FAYPAIRYL | C*12:03 | DAP3 | 0 | 0 | 0 | 0 | 0 | 0 |
| FVFPGELLL | C*12:03 | SLC1A5 | 0 | 0 | 0 | 0 | 0 | 0 |
| VPLPPKGRVL | C*12:03 | TMEM126B | 0 | 0 | 0 | 0 | 0 | 0 |
| APRHPSTNSL | B*42:01 | NDUFAF4 | 0 | 0 | **1** | 0 | 0 | 0 |
| RPKAQPTTL | B*42:01 | MED27 | 0 | 0 | **1** | 0 | 0 | 0 |
| TASPLVKSV | C*12:03 | ARHGAP11A | 0 | 0 | 0 | 0 | **1** | 0 |
| EYGHIPSF | A*24:02 | ARHGAP11A | **1** | **1** | 0 | 0 | 0 | 0 |
| TPSSRPASL | B*42:01 | UBL7 | **1** | **1** | 0 | 0 | 0 | 0 |
| KPQPRPQTL | C*12:03 | DYRK4 | **1** | **1** | **1** | 0 | 0 | 0 |
| KPRPPQGL | B*42:01 | MOGS | **1** | **1** | **1** | **1** | 0 | 0 |
| VPLTRVSGGAA | B*42:01 | SEMA4A | **1** | **1** | **1** | **1** | **1** | **1** |
|  |  |  |  |  |  |  |  |  |
|  |  |  |  |  |  |  |  |  |
| **Legend** |  |  |  |  |  |  |  |  |
| 24#1 | time after carfilzomib treatment/biological replicate | | | |  |  |  |  |
| -1 | significantly down-modulated | |  |  |  |  |  |  |
| 0 | not signifcant |  |  |  |  |  |  |  |
| 1 | significantly up-modulated | |  |  |  |  |  |  |

**Supplemental Table 2:**

**Summary of myeloma-associated peptides detected on MM.1S cells and their modulation upon carfilzomib-treatment**
