# Supplementary material for: Carfilzomib alters the HLA-presented peptidome of myeloma cells and impairs presentation of peptides with aromatic C-termini
Source: Blood Cancer J. 2016 Apr 8;6(4):e411–. doi: 10.1038/bcj.2016.14 (PMC4855252; doi:10.1038/bcj.2016.14)
Supplement: Supplementary Table 3 [file bcj201614x4.docx]

**Supplemental Table 3:**

**Summary of myeloma-associated peptides detected on U266 cells and their modulation upon carfilzomib-treatment**

| **Myeloma LiTAP** | **HLA** | **Source Protein** | **24#1** | **24#2** | **24#3** | **48#1** | **48#2** | **48#3** |
| --- | --- | --- | --- | --- | --- | --- | --- | --- |
| KAMEAASSL | B*07:02 | WHSC1 | -1 | -1 | -1 | -1 | -1 | -1 |
| KPKDPLKISL | B*07:02 | PPP2R3C | -1 | -1 | -1 | 0 | -1 | -1 |
| RVFPYSVFY | A*03:01 | NPC1 | -1 | 0 | 0 | -1 | -1 | -1 |
| SRGDFVVEY | C*07:02 | SETD8 | 0 | -1 | 0 | 0 | 0 | -1 |
| IIFDRPLLY | A*03:01 | DOLK | 0 | 0 | 0 | 0 | 0 | -1 |
| SVYSPVKKK | A*03:01 | NUPL2 | 0 | 0 | -1 | 0 | 0 | 0 |
| GEVQDLLVRL | B*40:01 | BAZ2A, BAZ2B | 0 | 0 | 0 | 0 | 0 | 0 |
| KAVNPGRSL | B*07:02 | BFAR | 0 | 0 | 0 | 0 | 0 | 0 |
| SPRLSLLYL | B*07:02 | BFAR | 0 | 0 | 0 | 0 | 0 | 0 |
| ILRDGITAGK | A*03:01 | BTN3A1, BTN3A2, BTN3A3 | 0 | 0 | 0 | 0 | 0 | 0 |
| RVAKTNSLR | A*03:01 | CDCA8 | 0 | 0 | 0 | 0 | 0 | 0 |
| TPAVGRLEV | B*07:02 | CDCA8 | 0 | 0 | 0 | 0 | 0 | 0 |
| SPALKRLDL | B*07:02 | COG1 | 0 | 0 | 0 | 0 | 0 | 0 |
| SPRQALTDF | B*07:02 | COG1 | 0 | 0 | 0 | 0 | 0 | 0 |
| AEQEIARLVL | B*40:01 | CREB3 | 0 | 0 | 0 | 0 | 0 | 0 |
| KILKPVKKK | A*03:01 | CSNK2A1, CSNK2A3,CSNK2A3 | 0 | 0 | 0 | 0 | 0 | 0 |
| ALWGRTTLK | A*03:01 | DAP3 | 0 | 0 | 0 | 0 | 0 | 0 |
| KPQPRPQTL | B*07:02 | DYRK4 | 0 | 0 | 0 | 0 | 0 | 0 |
| RVNKVIIGTK | A*03:01 | EIF2B2 | 0 | 0 | 0 | 0 | 0 | 0 |
| ILWETVPSM | A*02:01 | FNDC3B | 0 | 0 | 0 | 0 | 0 | 0 |
| RPGPPTRPL | B*07:02 | FNDC3B | 0 | 0 | 0 | 0 | 0 | 0 |
| SESLPVRTL | B*40:01 | FNDC3B | 0 | 0 | 0 | 0 | 0 | 0 |
| KLPLPLPPRL | A*02:01 | HSH2D | 0 | 0 | 0 | 0 | 0 | 0 |
| YLYITKVLK | A*03:01 | KDELR1, KDELR2 | 0 | 0 | 0 | 0 | 0 | 0 |
| KTEVHIRPK | A*03:01 | LAP3 | 0 | 0 | 0 | 0 | 0 | 0 |
| PELGPLPAL | B*40:01 | LRRC47 | 0 | 0 | 0 | 0 | 0 | 0 |
| RPKAQPTTL | B*07:02 | MED27 | 0 | 0 | 0 | 0 | 0 | 0 |
| FLWDEGFHQL | A*02:01 | MOGS | 0 | 0 | 0 | 0 | 0 | 0 |
| RPFHGWTSL | B*07:02 | MOGS | 0 | 0 | 0 | 0 | 0 | 0 |
| RQFWTRTKK | A*03:01 | MRPL55 | 0 | 0 | 0 | 0 | 0 | 0 |
| RPQLKGVVL | B*07:02 | MRPS12 | 0 | 0 | 0 | 0 | 0 | 0 |
| IESHPDNAL | B*40:01 | NAE1 | 0 | 0 | 0 | 0 | 0 | 0 |
| REEGTPLTL | B*40:01 | NOC2L | 0 | 0 | 0 | 0 | 0 | 0 |
| GEVAPSMFL | B*40:01 | NPC1 | 0 | 0 | 0 | 0 | 0 | 0 |
| SPYLRPLTL | B*07:02 | NUDT14 | 0 | 0 | 0 | 0 | 0 | 0 |
| KPSTKALVL | B*07:02 | RAD1 | 0 | 0 | 0 | 0 | 0 | 0 |
| KLSSLIILM | A*02:01 | SERPINH1 | 0 | 0 | 0 | 0 | 0 | 0 |
| LPPPPHVPL | B*07:02 | SLX1A | 0 | 0 | 0 | 0 | 0 | 0 |
| GETAFAFHL | B*40:01 | SLX1A | 0 | 0 | 0 | 0 | 0 | 0 |
| LLFPYILPPK | A*03:01 | SNX14 | 0 | 0 | 0 | 0 | 0 | 0 |
| IPAKPPVSF | B*07:02 | TXNDC11 | 0 | 0 | 0 | 0 | 0 | 0 |
| GPRPITQSEL | B*07:02 | UBL7 | 0 | 0 | 0 | 0 | 0 | 0 |
| TPSSRPASL | B*07:02 | UBL7 | 0 | 0 | 0 | 0 | 0 | 0 |
| RPRPPVLSV | B*07:02 | ZBTB21 | 0 | 0 | 0 | 0 | 0 | 0 |
| KEGLILPETL | B*40:01 | CREB3 | 1 | 1 | 0 | 0 | 0 | 0 |
| FAYPAIRYL | A*02:01 | DAP3 | 0 | 0 | 0 | 1 | 0 | 1 |
| FVFPGELLL | A*02:01 | SLC1A5 | 1 | 1 | 0 | 0 | 0 | 0 |
| APFQGDQRSL | B*07:02 | IRF9 | 0 | 1 | 1 | 0 | 1 | 0 |
| KPRPPQGL | B*07:02 | MOGS | 0 | 0 | 0 | 1 | 1 | 1 |
| APRHPSTNSLL | B*07:02 | NDUFAF4 | 0 | 1 | 0 | 1 | 1 | 0 |
| APRHPSTNSL | B*07:02 | NDUFAF4 | 0 | 1 | 0 | 1 | 1 | 0 |
|  |  |  |  |  |  |  |  |  |
|  |  |  |  |  |  |  |  |  |
| **Legend** |  |  |  |  |  |  |  |  |
| 24#1 | time after carfilzomib treatment/biological replicate | | | | | |  |  |
| -1 | significantly down-modulated | | | | |  |  |  |
| 0 | not signifcant | | | |  |  |  |  |
| 1 | significantly up-modulated | |  |  |  |  |  |  |
