# Supplementary figures and images for: Carfilzomib alters the HLA-presented peptidome of myeloma cells and impairs presentation of peptides with aromatic C-termini
Source: Blood Cancer J. 2016 Apr 8;6(4):e411–. doi: 10.1038/bcj.2016.14 (PMC4855252; doi:10.1038/bcj.2016.14)

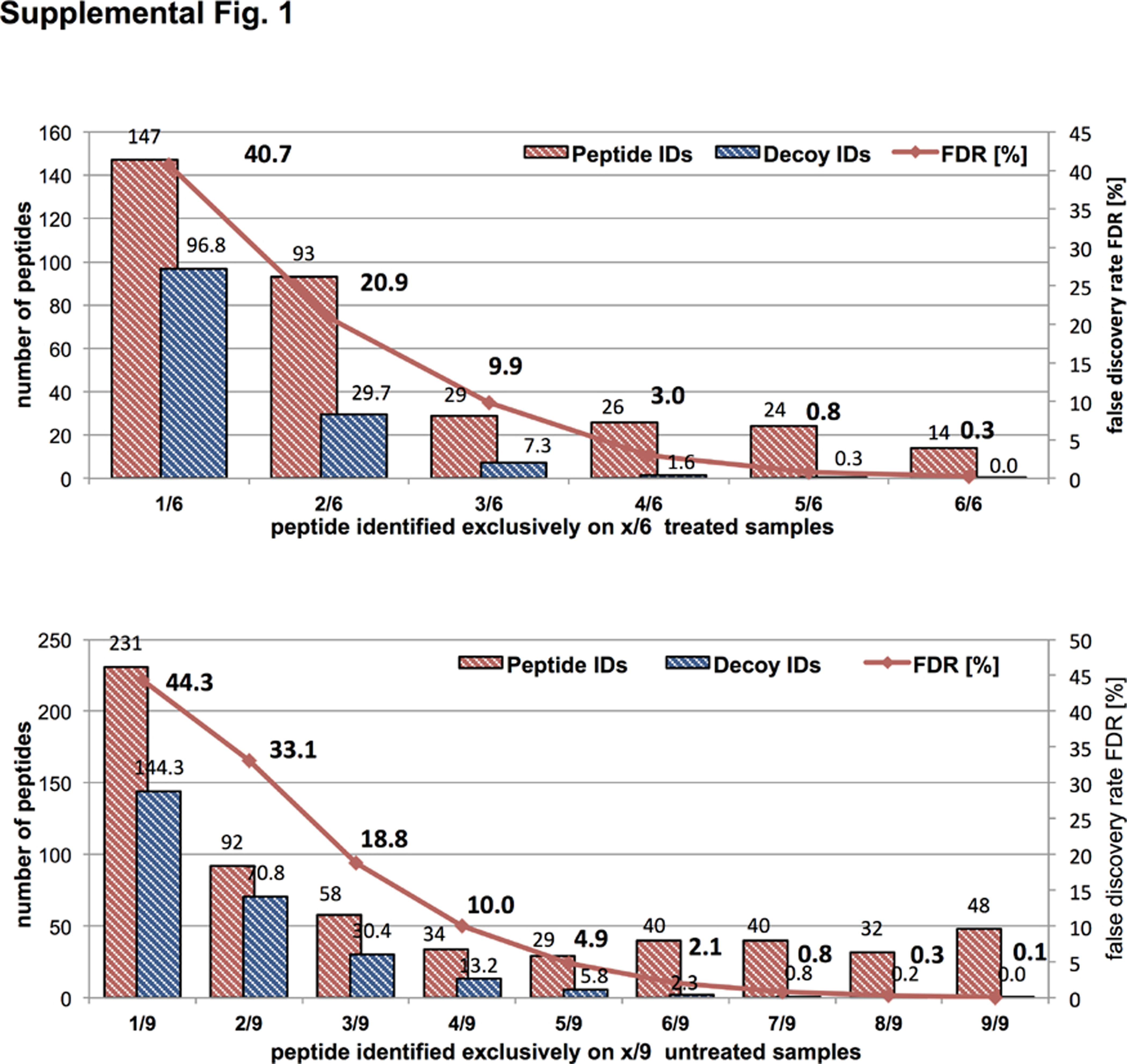

Supplement: Supplementary Figure 1 [file bcj201614x5.tif]

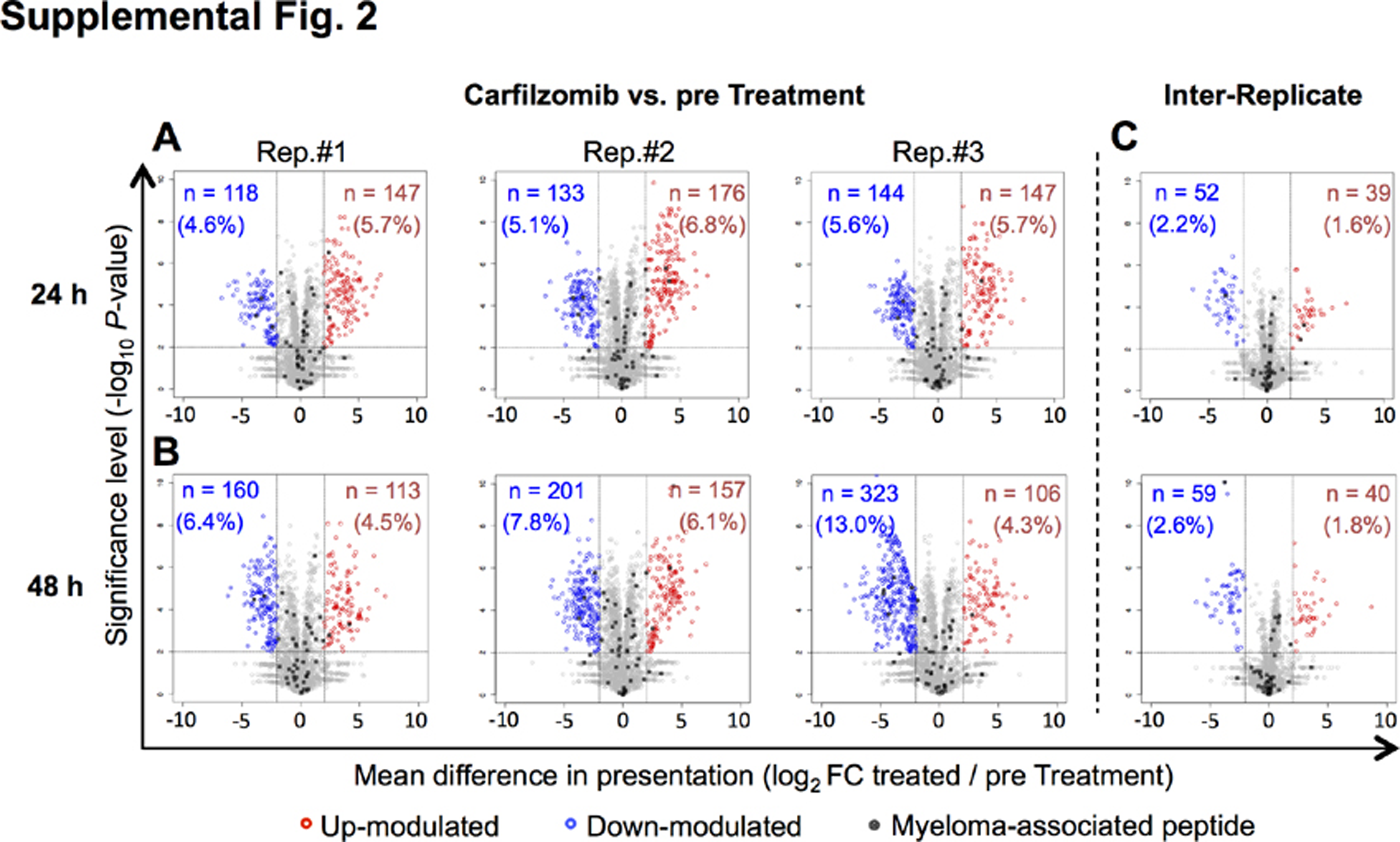

Supplement: Supplementary Figure 2 [file bcj201614x6.tif]

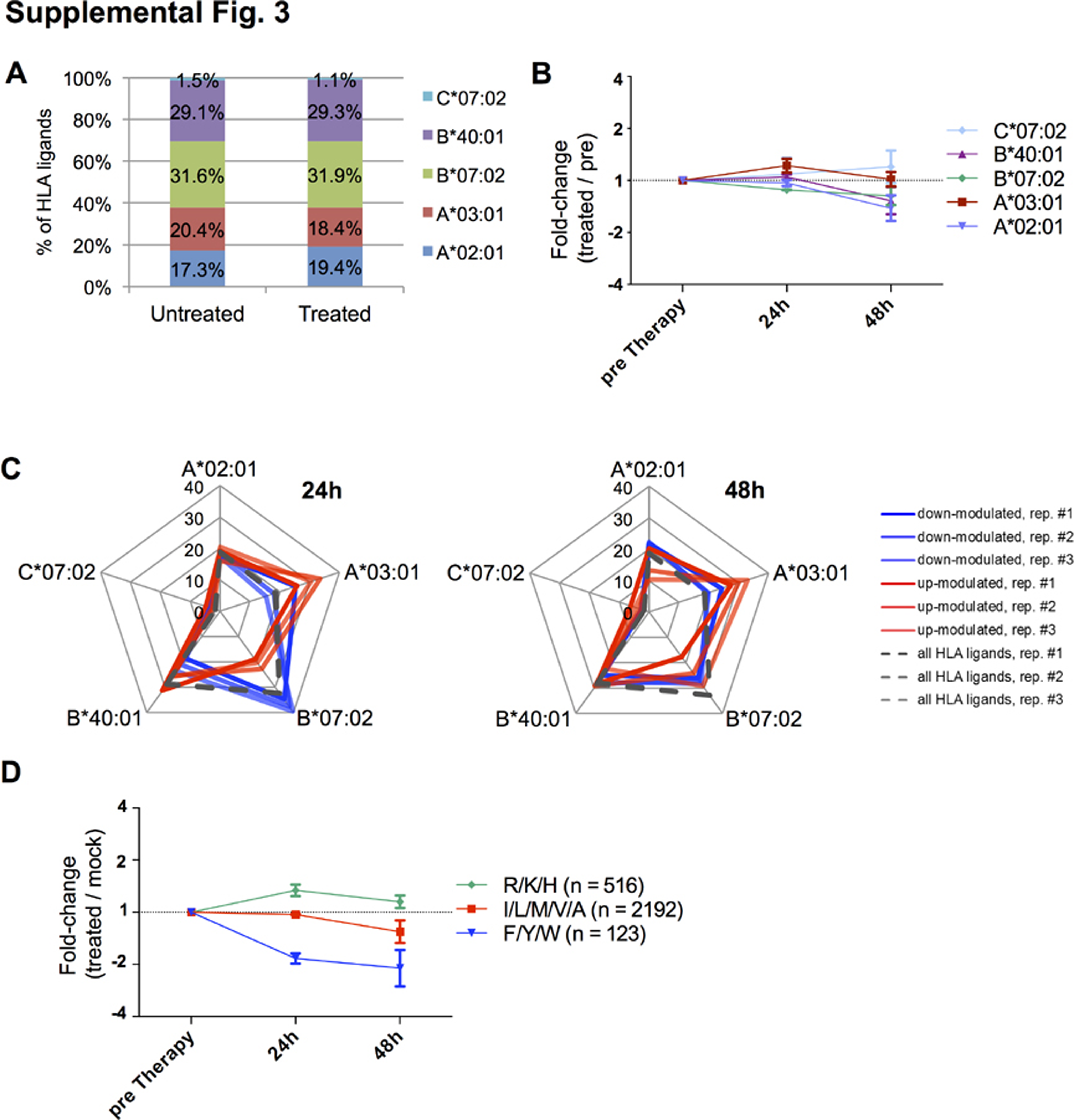

Supplement: Supplementary Figure 3 [file bcj201614x7.tif]
